# New-Onset Diabetes and Glucose Regulation Are Significant Determinants of Left Ventricular Hypertrophy in Renal Transplant Recipients

**DOI:** 10.1155/2015/293896

**Published:** 2015-04-07

**Authors:** Siren Sezer, Mehtap Erkmen Uyar, Emre Tutal, Zeynep Bal, Orhan Guliyev, Turan Colak, Efe Hasdemir, Mehmet Haberal

**Affiliations:** ^1^Department of Nephrology, Baskent University Medical School, 06490 Ankara, Turkey; ^2^Department of Internal Medicine, Baskent University Medical School, 06490 Ankara, Turkey; ^3^Department of Transplantation Surgery, Baskent University Medical School, 06490 Ankara, Turkey

## Abstract

*Background*. New-onset diabetes after transplantation (NODAT) is associated with decreased graft survival and an increased risk for cardiovascular disease. The objective of this study was to evaluate the risk factors for development of NODAT and its' relationship with arterial stiffness and left ventricular mass index (LVMI) in kidney transplant recipients. *Methods*. 159 kidney transplant recipients were selected from our transplantation center who underwent renal transplantation between years 2007 and 2010. *Results*. Among 159 patients, 57 (32.2%) patients were with NODAT who were significantly older than patients without diabetes (*P*: 0.0001). Patients with NODAT had significantly higher pulse wave velocity (PWv) (*P*: 0.033) and left ventricular mass index LVMI (*P*: 0.001) compared to patients without NODAT. Further analysis was done according to LVMI as follows: LVMI > 130 g/m^2^ (*n*: 57) and LVMI ≤ 130 g/m^2^ (*n*: 102). We observed higher office systolic and diastolic BP, serum trygliceride, glucose, creatinine, age, and HbA1c (*P*: 0.0001) levels in patients with LVMI > 130 g/m^2^. Linear regression analysis revealed that HbA1c was the major determinant of LVMI (*P*: 0.026, *β*: 0.361). *Conclusions*. HbA1c is the major determinant of LVMI, so strict control of serum glucose levels is essential for preventing cardiovascular disease in patients with NODAT.

## 1. Introduction

New-onset diabetes after transplantation (NODAT) is a serious complication associated with decreased graft and patient survival as well as an increased risk for cardiovascular disease [1, 2]. New-onset diabetes after transplantation most frequently develops in the early posttransplant period, usually within the first year [2], and has additionally been linked with all-cause mortality, increased susceptibility to infections, acute rejection, chronic graft dysfunction, and decreased quality of life [3–6].

The precise incidence of NODAT is difficult to determine, with a widely dispersed reported incidence between 2% and 50% [7], due to the lack of a uniform definition, varying immunosuppressive regimens, and variable duration of follow-up [1, 8]. Multiple risk factors for NODAT have been identified, including advanced age at transplantation, ethnicity, and obesity, a family history of diabetes, impaired glucose tolerance before transplantation, CMV infection, and drugs [9]. The evaluation of all these factors and determination of the treatment modalities will be a guide in the prevention of NODAT complications.

Presence of left ventricular hypertrophy and arterial stiffness are independent determinants of cardiovascular disease in patients with end-stage renal disease [10]. Left ventricular hypertrophy might have negative impact on development of myocardial ischemia, arrhythmias, congestive heart failure, and sudden death [11, 12]. Renal transplantation generally leads to regression of left ventricular hypertrophy though it may persist or develop de novo in some patients [12]. In previous literature, a higher proportion of prediabetic states and NODAT were shown to be related with increasing left ventricular mass after transplantation [11, 13, 14].

Arterial stiffness is an independent predictor of cardiovascular events in patients with hypertension [15–17], ESRD, and diabetes mellitus [18]. Pulse wave velocity is an easy (PWv), noninvasive, and repeatable tool that could be used to evaluate the degree of atherosclerosis and arterial stiffness [19]. A number of studies revealed that restoration of renal function after renal transplantation could partially improve increased arterial stiffness [20, 21]. On the other hand, it has also been shown that renal transplant recipients with glucose intolerance had increased PWv, suggesting that glucose intolerance may induce atherosclerosis [4, 5, 22].

The objective of this study was to evaluate the risk factors for the development of NODAT and its relationship with arterial stiffness and left ventricular hypertrophy in kidney transplant recipients.

## 2. Methods

### 2.1. Selection of the Population

Between years 2007 and 2010, 63 (23.59%) kidney transplant patients out of 267 were diagnosed to have NODAT in our center. Among these, 159 kidney transplant recipients were selected according to the following exclusion criteria: (1) irregular drug usage or patient incompliance, (2) lack of regular follow-up data, (3) pretransplant diabetes mellitus history, (4) bone marrow transplant or other solid organs before or at the time of transplantation (including previous kidney transplantation), (5) malign disease, rheumatologic or chronic inflammatory disease of unknown origin, systemic vasculitis history, (6) acute rejection periods after the first year of transplantation, (7) unstable cardiac disease including heart failure (ejection fraction < 50%) and/or ischemic heart disease history (myocardial infarction, need for cardiac revascularization), (8) atrial fibrillation or elevated heart rate (>100 beats/min), (9) coronary bypass before or after transplantation, (10) transiently elevated fasting plasma glucose (FPG) or diabetic blood glucose profile during the first 3 posttransplant months, (11) graft failure (glomerular filtration (GFR) rate < 30 mL/min), and (12) history of peritoneal dialysis before transplantation.

New-onset diabetes after transplantation was defined as a fasting plasma glucose (FPG) level ≥ 126 mg/dL (7 mmol/L) or symptoms of diabetes plus casual plasma glucose concentrations ≥200 mg/dL (11.1 mmol/L), confirmed by repeat testing on a different day [23]. According to these criteria, 63 patients were diagnosed as NODAT between years 2007 and 2010 but after the exclusion criteria of our study 57 patients with and 102 patients without NODAT were included in the study.

### 2.2. Clinical and Biochemical Measurements

All patients' data were recorded from clinical charts. Visits in outpatient clinic were organized as follows: three visits per week during the first 2 weeks; two visits per week until Day 60; weekly visits until Day 120; monthly visits during the first year; one visit every other month during the second year; and four visits per year thereafter until death or end-stage renal disease (i.e., dialysis or retransplantation). The following parameters were collected: (1) age, (2) gender, (3) posttransplantation duration, (4) pretransplant hemodialysis duration, (5) acute rejection episodes, (6) use of statins, ace inhibitor (ACE) or angiotensin receptor blocker (ARB), (7) immunosuppressive treatment (mycophenolate, cyclosporine, tacrolimus, and sirolimus use), (8) pretransplant lipid profile (values in the last month before transplantation), (9) posttransplant lipid profile (mean value), (10) FPG and HbA1c levels (mean value), (11) office blood pressure measurements, (12) hemoglobin, calcium, phosphorus, albumin and parathyroid hormone levels, (13) creatinine and eGFR (MDRD equation), and (14) cytomegalovirus infection history.

Mean values were arithmetic means of each parameter that were collected from patient charts at monthly basis after the first posttransplant of 6 months while other parameters were collected as single values at study inclusion.

Maintenance immunosuppressive treatment included prednisone with a gradual tapering and mycophenolate mofetil or sodium associated with cyclosporine, tacrolimus, or sirolimus in most patients. Target through levels at 3 months were 150–250 ng/mL for cyclosporine and 8–12 ng/mL for tacrolimus and sirolimus. Steroids were tapered: 500 mg methylprednisolone intravenous (iv) on Day 0, 250 mg iv on Day 1, and 100 mg iv on Day 2 and 50 mg prednisolone perorally (po) from Day 3 to Day 6, 0.5 mg/kg BW po from Day 7 with a reduction by 5 mg every 2 weeks until 15 mg/day, and then 2.5 mg every 2 weeks until a maintenance dose 5 mg was achieved. All patients were under 5 mg prednisolone treatment within the maintenance immunosuppressive regimen 6 months after transplantation. Antidiabetic treatment modalities (diet and lifestyle changes, oral antidiabetic drugs, or insulin) were also recorded for patients with NODAT.

Body compositions of all patients were analyzed by using the body composition analyzer (Tanita BC-420MA). Fat mass, fat free mass, muscle mass, visceral fat mass, and body mass index were calculated for each patient.

All patients underwent echocardiographic examinations (Siemens Acuson C256, Mountain View, CA, 2000 with 3V2c transducer probe) by the same operator and left ventricular mass was calculated according to the Devereux formula and indexed to body surface area to give left ventricular mass index (LVMI) (g/m^2^). Left ventricular mass index values greater than 130 g/m^2^ (*n*: 57) were defined as high left ventricular mass.

Pulse wave velocity (PWv) is defined as the velocity of the arterial pulse for moving along the vessel wall. Pulse wave velocity along the aorta was measured by using two ultrasound or pressure sensitive transducers fixed transcutaneously over the course of a pair of arteries separated by a known distance: the femoral and right common carotid arteries. PWv was calculated from measurements of pulse transit time and the distance, according to the following formula: PWv (m/s) = distance (m)/transit time (s). Measurement of PWv values was con-ducted after abstinence from caffeine or smoking and after an overnight fast without intake of antihypertensive drugs. PWv was determined by using the SphygmoCor CvMs V9 system and values > 7 m/s were defined as increased.

### 2.3. Statistical Analysis

Statistical analyses were performed by using a SPSS software (Statistical Package for the Social Sciences, version 11.0, SSPS Inc., Chicago, IL, USA). Subjects were grouped according to the presence of NODAT as diabetic (*n*: 57) and nondiabetic (*n*: 102) patient groups and according to presence of left ventricular hypertrophy as increased LVMI (*n*: 57) and normal LVMI (*n*: 102) patient groups. Differences between these groups were analyzed separately. Patients with NODAT were also divided into three groups according to their treatment as patients with diet and life-style changes (*n* = 29), oral antidiabetics (*n* = 5), and insulin (*n* = 23) for further analysis.

Normality of data was analyzed by using a Kolmogorov-Smirnov test. All numerical variables with normal distribution were expressed as the means ± standard deviations (SD), while variables with skew distribution were expressed as medians and interquartile range (IR). Categorical variables were expressed as percentages and compared by chi-square test. Normally distributed numeric variables were analyzed by independent samples *t* or one-way ANOVA (post hoc Tukey) tests according to distribution normality. Skew distributed numeric variables were compared using the Mann-Whitney *U* and Kruskal-Wallis tests according to distribution normality. Spearman and Pearson Correlation tests were used for correlation analyses. Linear regression analysis was performed to assess the major determinant of high LVMI between correlated variables. A *P* value < 0.05 was considered as statistically significant.

## 3. Results 

### 3.1. NODAT and Biochemical Parameters

Among 159 patients, 57 (32.2%) patients were with NODAT who were significantly older than patients without diabetes (43.2 ± 10.7 versus 37.0 ± 10.3 years, *P*: 0.0001, Table 1).

There was no significant difference between groups in means of biochemical parameters except posttransplant triglyceride levels which were slightly higher in NODAT group (198.2 ± 93.1 versus 164.3 ± 101.9 mg/dL, *P*: 0.03, Table 1). FPG and HbA1c levels were also higher in NODAT group as expected.

### 3.2. NODAT and Anthropometric Measurements

Patients with NODAT also had significantly higher BMI than patients without NODAT (28.5 ± 5.4 versus 24.7 ± 4.1 kg/m^2^, *P*: 0.0001). In body composition analysis, fat mass (20.4 ± 9.0 versus 15.3 ± 8.2 kg, *P*: 0.001) and fat free mass (8.5 ± 3.7 versus 5.9 ± 4.2 kg, *P*: 0.001) were significantly higher in patients with NODAT (Table 1).

There was no statistically significant difference between patients with and without NODAT by means of using statins, ACE inhibitors, or ARB (*P* > 0.05) (Table 1). There was no statistically significant difference in CMV infection history between patients with and without NODAT (14 (24.5%) versus 28 (26.7%), resp.) (*P*: 0.861) (Table 1).

### 3.3. NODAT and Cardiovascular Indices

We observed higher office systolic BP level (134.4 ± 24.5 versus 122.0 ± 45.1 mmHg, *P*: 0.023) in patients with NODAT. The percentage of patients with high LVMI (>130 g/m^2^) was significantly higher in patients with NODAT (63.2% versus 21.6%, *P*: 0.0001, Table 1). Patients with NODAT had significantly higher PWv (7.37 ± 1.9 versus 6.68 ± 2.16 m/s *P*: 0.033) and LVMI (151.9 ± 47.9 versus 125.9 ± 45.05 g/m^2^, *P*: 0.001) compared to patients without NODAT (Table 1).

### 3.4. NODAT and LVMI Groups

Further analysis was done after dividing study population into two groups according to LVMI as follows: LVMI > 130 g/m^2^ (*n*: 57) and LVMI ≤ 130 g/m^2^ (*n*: 102, Table 2). We observed higher office systolic (143.9 ± 17.1 mmHg versus 130.7 ± 21.9 mmHg, *P*: 0.0001) and diastolic (87.9 ± 10.4 mmHg versus 82.3 ± 14.7 mmHg, *P*: 0.01) BP, serum triglyceride (193.9 ± 88.2 versus 164.4 ± 105.3 mg/dL, *P*: 0.047), glucose (119.1 ± 52.2 versus 93.8 ± 22.0 mg/dL, *P*: 0.0001), age (*P*: 0.007), and HbA1c (7.4 ± 1.6% versus 6.3 ± 1.2%, *P*: 0.0001) levels in patients with LVMI >130 g/m^2^ (Table 2). Serum creatinine levels were significantly higher in patients with LVMI >130 g/m^2^ (1.4 ± 0.5 versus 1.2 ± 0.5 mg/dL, *P*: 0.049). Serum calcium, phosphorus, parathyroid hormone (PTH), albumin, hemoglobin, and pretransplantation lipid parameters were similar in both groups (Table 2). Body composition analysis and body compartments of both LVMI groups were similar (Table 2). The percentage of patients with PWv > 7 m/s tended to be higher in patients with higher LVMI but this difference was not statistically significant (34.8% g/m^2^ versus 30.7 g/m^2^, *P*: 0.340).

### 3.5. NODAT Treatment Groups

There was no significant difference in LVMI, PWv, or HbA1c levels between NODAT treatment groups (*P* > 0.05, Table 3). Only LVMI of patients under insulin therapy was significantly higher than patients without NODAT (158.11 ± 66.41 versus 125.9 ± 45.05, *P*: 0.05). When we analyzed relationship between glucose regulation and LVMI when the patients were grouped according to HbA1c levels we observed that LVMI was still significantly higher in NODAT patients with low HbA1c levels (<6.5% HbA1c levels, *n*: 33) than patients without NODAT (147.4 ± 32.31 versus 134.6 ± 50.02 g/m^2^, *P*: 0.013).

**Figure 1:**
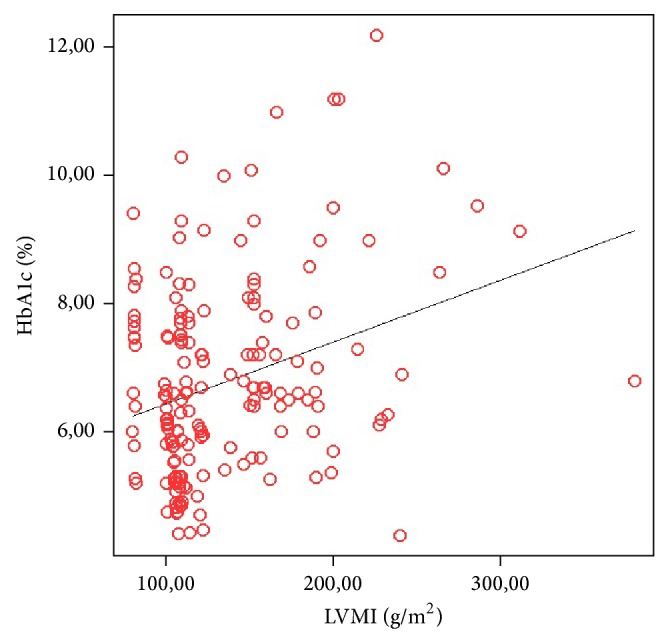
Linear regression analysis of factors affecting left ventricular mass revealed that HbA1c was the major determinant of LVMI (*P*: 0.026, *β*: 0.361).

Linear regression analysis of factors affecting left ventricular mass revealed that HbA1c was the major determinant of LVMI (*P*: 0.026, *β*: 0.361) (Figure 1).

## 4. Discussion

New-onset diabetes after transplantation is a serious metabolic complication that has been reported to develop in 2–53% of all solid organ transplants and 4 to 25% of renal transplant recipients [24–26]. These wide variations in reported incidences are due to lack of uniform definition used, presence of variable risk factors in population under study, method of detection, and duration of follow-up [27]. The incidence of NODAT in our study group was 23.59% consistent with previous literature.

Posttransplant hyperglycemia usually develops in patients with a high cardiovascular risk profile; older recipients with higher BMI; or those with insulin resistance before transplantation [28]. Older age of recipients is considered as the most important risk factor for NODAT [8]. Consistent with this data, our patients with NODAT were significantly older and had higher BMI, fat mass, and fat free mass compared to patients without NODAT. Chakkera et al. observed that pretransplant elevated serum triglyceride level was an important risk factor for NODAT development [29]. Hypertriglyceridemia is known to be associated with insulin resistance and atherosclerosis in previous studies [30]. In our study, patients with NODAT had nonsignificantly higher pretransplant serum triglyceride levels than patients without diabetes. In our subjects, longer dialysis before transplantation also appeared to confer a higher risk of diabetes after kidney transplantation.

The association between the use of tacrolimus and the development of NODAT has been clearly established previously [1, 31]. However in our study population there was no significant difference between immune suppressive regimes in terms of the frequency of NODAT. This may be because of the relatively small number of patients included that did not reveal a statistical significance, though there was a higher tendency in the tacrolimus group to develop NODAT.

Although steroids are known to have a strong diabetogenic effect [32, 33], we did not observe any association with NODAT. This finding could be explained by an intentional decrease in the dose of corticosteroids when diabetes is recognized as in our transplantation outpatient clinic and the low maintenance dose of steroids used by the patients. This clinical practice was observed in a previous study with steroid dose decline [34]. As we excluded patients with acute rejection episodes, our patients had not received any pulse steroid regimen. There is also some evidence about the influence of nonimmunosuppressive drugs such as statins and antihypertensives on development of NODAT [35, 36]. In contrast to these studies, we did not find any relationship between NODAT and the use of statins, ACE inhibitors, or ARB in our study group.

Left ventricular hypertrophy, one of the structural alterations involved in diabetic cardiomyopathy [37–39], has also been associated with abnormal glucose tolerance in several epidemiological investigations [40–42]. We observed that the LVMI was significantly higher among patients who developed NODAT. The influence of glucose intolerance, insulin resistance, and metabolic syndrome on the LVMI has been reported by some previous remarkable epidemiologic studies [43, 44], in hypertensive nondiabetic [45, 46] and diabetic nontransplanted subjects [47, 48]. In this study, when we compared patients according to their LVMI, we observed higher office systolic and diastolic blood pressure, serum triglyceride, creatinine, glucose, and HbA1c levels in patients with LVMI >130 g/m^2^. Patients with a high LVMI showed worse renal function, associated with greater proportion of subjects with diabetes mellitus after transplantation. Left ventricular hypertrophy has been strongly linked to renal functional impairment [11, 12, 49, 50]. In this study, higher LVMI was associated with worse renal function, having significantly higher creatinine levels and lower GFR than patients with LVMI ≤130 g/m^2^. In the context of both left ventricular hypertrophy and NODAT, markers of increased oxidative stress are known to be elevated, leading to graft failure [51].

In diabetes, nonenzymatic glycation of proteins and formation of advanced glycation endproducts have the potential to quench nitric oxide and then diminish the vasodilatory capacity of the peripheral muscular arteries [52, 53]. Reduced nitric oxide availability may cause vasoconstriction and alter growth of vascular muscle, as well as producing cellular injury in prolonged hyperglycemia [54]. The formation of advanced glycation endproducts on collagen is enhanced within the arterial wall and may also contribute to vascular injury [55]. Thus prolonged hyperglycemia can modify the timing and magnitude of the pulse wave reflection to augment systolic load of the left ventricle [54]. The impaired systolic loading condition of the left ventricle may cause the heart to adapt to muscular hypertrophy and may increase the ratio of left ventricular weight to body weight, an indicator of cardiac hypertrophy [54]. Accordingly, regression analysis of risk factors for the development of LVMI revealed that HbA1c was the major determinant of LVMI, indicating the importance of serum glucose control. All of these findings suggest that glucose homeostasis plays pivotal roles in the evolution of ventricular mass after renal transplantation. In the diabetic population, HbA1c level is a validated and reliable marker for glycemic control and for predicting morbidity and mortality [56]. But even in NODAT patients with good glycemic control and low HbA1c levels (<6.5% HbA1c levels) LVMI was still significantly greater to those without NODAT. Our result showed that though well controlled the presence of NODAT should be accepted not only as a primary risk factor but also as a direct prometer of the development of left ventricular hypertrophy in renal transplant recipients.

High pulse wave velocity is a universal marker of aortic stiffness and the link between pulse pressure and NODAT is unknown [7]. Aortic stiffness leading to microvascular injury within the pancreas circulation (leading to impaired insulin secretion) may be one of the mechanisms of NODAT [57]. Interestingly, hypertension was found to be a risk factor for the development of diabetes mellitus in the general population [58]. This association suggests a link between vascular damage and the onset of diabetes [59]. Ultrastructural alterations of vascular pancreatic islets with loss of endothelial cell homeostasis have been suggested to play a key role in the pathogenesis of beta-cell dysfunction [60]. Our data showed that NODAT is related with increased PWv. Hyperglycemia and associated relative deficiency of insulin secretion may negatively modulate a wide array of cardiovascular risk factors, including redox imbalance and increased oxidative stress [61]. In NODAT, the formation of advanced glycation endproducts is also enhanced within the arterial wall and may contribute to vascular injury [55]. New-onset diabetes after transplantation and arterial stiffness have a bimodal relationship in a cause and effect manner. Presence of NODAT, hypertension, older age, and longer pretransplant dialysis duration were all related with increased PWv as in previous literature [7, 8, 28, 62]. All these comorbidities aggregate endothelial dysfunction and enhance atherosclerosis and therefore increase arterial stiffness.

Our study has several limitations. It is a cross sectional study, and our findings need to be confirmed in large long-term prospective studies. Pretransplantation echocardiographic and arterial pulse wave evaluations of patients were absent, so we were not able to discuss the initiation or the progression of LVMI or arterial stiffness. Pretransplant OGTT was not routinely assessed at our center. There are other less consistent risk factors linked to development of NODAT, like HLA phenotype, genetic polymorphisms of interleukins, and donor characteristics, through levels of CNIs. These issues were not addressed in our study. Patients' antihypertensive medication was recorded according to the type of the class and we could not assess the influence of the type and dose because of the small number of patients.

The current study confirmed the association of traditional risk factors including age, presence of obesity, and long dialysis duration with the development of NODAT. Patients with NODAT should be accepted as a high risk population with higher prevalence of left ventricular hypertrophy and vascular stiffness. Prevention of NODAT related cardiovascular morbidity may be accomplished with early detection of this metabolic disorder with corresponding therapeutic interventions such as change in lifestyle, weight loss, selection of an appropriate immunosuppressive regimen, and use of antidiabetic drugs. Thus, regular screening of NODAT and initiation of proper treatment and better metabolic control at earliest possible should be the integral part of overall renal transplant management. Relevance and therapeutic consequences must be determined in large-scale prospective studies.

## Figures and Tables

**Table 1:** The clinical and biochemical parameters of patients according to NODAT groups.

	Patients with NODAT (*n*: 57)	Patients without NODAT (*n*: 102)	*P* value
Age (years)	*43.2 ± 10.7 *	*37.0 ± 10.3 *	*0.0001 *
Gender (M)	35 (60.3%)	83 (68.0%)	0.198
Dialysis duration (months)	*56.5 ± 59.1 *	*32.1 ± 39.0 *	*0.010 *
Posttransplant duration (months)	50.3 ± 59.9	57.4 ± 54.5	0.393
Pretx T. Chol (mg/dL)	173.8 ± 45.9	171.3 ± 50.9	0.296
Pretx LDL Chol (mg/dL)	92.6 ± 33.8	97.3 ± 34.3	0.506
Pretx HDL Chol (mg/dL)	41.1 ± 14.5	42.7 ± 17.9	0.457
Pretx triglyceride (mg/dL)	185.6 ± 125.14	158.4 ± 86.4	0.139
GFR (mL/min)	68.68 ± 23.08	69.44 ± 24.9	0.841
Creatinine (mg/dL)	1.2 ± 0.5	1.3 ± 0.5	0.472
Albumin (g/L)	4.0 ± 0.4	4.1 ± 0.3	0.169
Hemoglobin (g/dL)	*13.2 ± 1.9 *	*13.2 ± 1.9 *	0.832
T. Chol (mg/dL)	215.9 ± 66.3	209.0 ± 46.4	0.483
LDL Chol (mg/dL)	116.4 ± 40.7	119.7 ± 34.3	0.599
HDL Chol (mg/dL)	47.7 ± 15.6	49.0 ± 13.4	0.605
Triglyceride (mg/dL)	*198.2 ± 93.1 *	*164.3 ± 101.9 *	*0.030 *
Calcium (mg/dL)	9.4 ± 0.5	9.2 ± 0.6	0.475
Phosphorus (mg/dL)	3.1 ± 0.7	3.2 ± 0.7	0.248
PTH (pcg/mL)	193.4 ± 24.4	148.8 ± 96.9	0.092
Glucose (mg/dL)	*136.2 ± 52.2 *	*87.3 ± 9.2 *	*0.0001 *
Office SBP (mmHg)	*134.4 ± 24.5 *	*122.0 ± 45.1 *	*0.023 *
Office DBP (mmHg)	83.3 ± 16.0	76.9 ± 28.5	0.065
HbA1c	*7.39 ± 1.33 *	*5.27 ± 1.131 *	*0.0001 *
BMI (kg/m^2^)	*28.5 ± 5.4 *	*24.7 ± 4.1 *	*0.0001 *
Fat mass (kg)	*20.4 ± 9.0 *	*15.3 ± 8.2 *	*0.001 *
Fat free mass (kg)	55.5 ± 12.4	54.7 ± 9.6	0.707
Visceral fat mass (kg)	*8.5 ± 3.7 *	*5.9 ± 4.2 *	*0.001 *
Muscle mass (kg)	53.6 ± 9.7	52.0 ± 9.2	0.341
PWv (m/s)	*7.37 ± 1.9 *	*6.68 ± 2.16 *	*0.033 *
PWv >7 m/s	*37 (63.8%) *	*29 (23.8%) *	*0.0001 *
LVMI (g/m^2^)	*151.9 ± 47.9 *	*125.9 ± 45.05 *	*0.001 *
LVMI >130 g/m^2^	*37 (63.8%) *	*29 (23.8%) *	*0.001 *
ACEI	7 (13.5%)	21 (17.5%)	0.339
AT II	12 (23.1%)	31 (25.8%)	0.429
Statin	12 (23.1%)	26 (21.7%)	0.492
Tacrolimus	28 (54.9%)	44 (41.9%)	0.306
Cyclosporin-A	13 (25.5%)	33 (31.4%)	0.149
Sirolimus	10 (19.6%)	28 (26.7%)	0.306
CMV infection	14 (24.5%)	28 (26.7%)	0.861

**Table 2:** The clinical and biochemical parameters of patients according to LVMI groups.

	LVMI > 130 g/m^2^ (*n*: 57)	LVMI ≤ 130 g/m^2^ (*n*: 102)	*P* value
Age (years)	*42.2 ± 10.6 *	*37.2 ± 10.5 *	*0.002 *
Gender (M)	44 (66.7%)	74 (64.9%)	0.871
Dialysis duration (months)	47.0 ± 49.5	35.8 ± 46.3	0.165
Posttransplant duration (months)	54.6 ± 52.2	55.4 ± 53.7	0.930
GFR (mL/min)	*63.23 ± 23.87 *	*72.65 ± 24.01 *	*0.012 *
Creatinine (mg/dL)	*1.4 ± 0.5 *	*1.2 ± 0.5 *	*0.049 *
Albumin (g/L)	4.0 ± 0.4	4.1 ± 0.3	0.43
Hemoglobin (g/dL)	13.2 ± 2.0	13.2 ± 1.9	0.845
T. Chol (mg/dL)	213.9 ± 52.4	209.7 ± 54.1	0.610
LDL Chol (mg/dL)	120.0 ± 40.0	117.9 ± 34.3	0.723
HDL Chol (mg/dL)	47.6 ± 14.2	49.2 ± 14.0	0.462
Triglyceride (mg/dL)	*193.9 ± 88.2 *	*164.4 ± 105.3 *	*0.047 *
Calcium (mg/dL)	9.4 ± 0.6	9.2 ± 0.6	0.065
Phosphorus (mg/dL)	3.1 ± 0.5	3.2 ± 0.7	0.301
PTH (pcg/mL)	170.8 ± 115.1	157.2 ± 131.5	0.492
Glucose (mg/dL)	*119.1 ± 52.2 *	*93.8 ± 22.0 *	*0.0001 *
Office SBP (mmHg)	*143.9 ± 17.1 *	*130.7 ± 21.9 *	*0.0001 *
Office DBP (mmHg)	*87.9 ± 10.4 *	*82.3 ± 14.7 *	*0.01 *
HbA1c	*7.4 ± 1.6 *	*6.3 ± 1.2 *	*0.0001 *
BMI (kg/m^2^)	26.9 ± 4.4	25.3 ± 4.9	0.102
Fat mass (kg)	17.7 ± 8.1	16.2 ± 9.0	0.310
Fat free mass (kg)	55.8 ± 12.3	54.5 ± 9.4	0.532
Visceral fat mass (kg)	7.25 ± 3.8	6.42 ± 4.43	0.237
Muscle mass (kg)	53.7 ± 9.9	51.8 ± 9.0	0.244

**Table 3:** HbA1c, PWv, and LVMI values of patients according to NODAT treatment modalities.

	Patients without NODAT (*n* = 102)	Diet and life-style changes (*n* = 29)	Oral antidiabetics (*n* = 5)	Insulin (*n* = 23)	*P* value
HbA1c (%)	5.27 ± 1.131	7.52 ± 0.77	7.20 ± 0.14	8.03 ± 1.46	0.273
PWv (m/s)	6.68 ± 2.16	9.25 ± 1.54	8.58 ± 0.78	9.19 ± 1.54	0.557
LVMI (g/m^2^)	***125.9 ± 45.05*** ^*^	*143.80 ± 31.84 *	*170.54 ± 18.46 *	***158.11 ± 66.41*** ^*^	***0.05*** ^*^

^*^ *P* value for LVMI between patients without NODAT and patients with NODAT under insulin therapy.
